# The Predictive Value of PD-L1 Expression Level in Evaluating the Cost-Effectiveness of Atezolizumab/Pembrolizumab

**DOI:** 10.3389/fonc.2022.857452

**Published:** 2022-04-22

**Authors:** Shen Lin, Yiyuan Li, Dian Gu, Shaohong Luo, Xiaoting Huang, Liangliang Dong, Xiongwei Xu, Peili Lin, Xiuhua Weng

**Affiliations:** ^1^ Department of Pharmacy, The First Affiliated Hospital of Fujian Medical University, Fuzhou, China; ^2^ Institute for Health and Aging, University of California, San Francisco, San Francisco, CA, United States

**Keywords:** cost-effectiveness analysis, budgetary impact analysis, PD-L1 expression level, non-small cell lung cancer, predictor, atezolizumab, pembrolizumab

## Abstract

**Objective:**

Recently, the significant improvement of atezolizumab and pembrolizumab over chemotherapy for treatment-naïve stage IV non-small cell lung cancer (NSCLC) has been demonstrated, but the cost-effectiveness of these regimens remains unknown.

**Methods:**

A Markov model was adapted from the US healthcare perspective to assess the cost-effectiveness of atezolizumab, pembrolizumab, and chemotherapy in treatment-naïve NSCLC. Pseudo-individual patient data were generated from digitized Kaplan–Meier curves. Direct medical costs and utility values were sourced from the database and literature. Quality-adjusted life-years (QALYs), total costs, and incremental cost-effectiveness ratios (ICERs) were computed. Sensitivity analyses and budgetary impact analyses were calculated.

**Results:**

In any and high programmed cell death 1-ligand 1 (PD-L1) expression populations, with chemotherapy, atezolizumab provided ICERs of $234,990 and $130,804 per QALY, while pembrolizumab yielded ICERs of $424,797 and $140,873 per QALY. The ICER of atezolizumab vs. pembrolizumab was $56,635 and $115,511.82 in any and high PD-L1 expression population, respectively. The critical drivers of ICERs included the cost of atezolizumab and pembrolizumab. The accumulated incremental budgetary impact of atezolizumab vs. chemotherapy increased to approximately $39.1 million in high PD-L1 expression patients over 5 years.

**Conclusions:**

In the high PD-L1 expression population, both atezolizumab and pembrolizumab were cost-effective for stage IV NSCLC compared to chemotherapy, which is contrary to that in any PD-L1 expression population. Atezolizumab shows a higher acceptability in both populations. Treating with immune checkpoint inhibitors (ICIs) has a substantial budgetary impact on the medical burden. The PD-L1 expression level has the potential to be a predictor for the economics of ICIs.

## Introduction

The latest Global Burden of Disease Study revealed that lung cancer is one of the leading causes of non-communicable disease burden worldwide (1), with 2.1 million new cases and 1.8 million deaths worldwide. Up to 61% of patients with non-small cell lung cancer (NSCLC) are metastatic or in the advanced stage at the time of diagnosis, which has a lower 5-year survival rate of 18% (2). The use of immune checkpoint inhibitors (ICIs) has brought great hope to patients. It should be clear that the clinical guidelines recommend that physicians consider the PD-L1 expression level of patients when using ICI monotherapy (3).

The programmed cell death 1-ligand 1 (PD-L1) expression has been identified as the major biomarker of the patients’ response to PD-1 and PD-L1 inhibitors. Recently, a phase III clinical trial, IMpower-110, has proved that PD-L1 inhibitor atezolizumab monotherapy provided longer overall survival (OS) and progression-free survival (PFS) in comparison with standard chemotherapy in the first-line treatment of metastatic NSCLC (4), and a better benefit was observed in a higher PD-L1 expression level subgroup (≥50%). The finding of the relation between PD-L1 expression and efficacy was in accord with another clinical trial, KEYNOTE-042, which indicated that more apparent survival benefits were found in the higher PD-L1 expression level population treated with pembrolizumab monotherapy (5).

Although the efficacy of ICIs has been confirmed, the high cost hinders their broad prescription in clinical practice. Since the efficacy of ICIs can be judged by PD-L1 expression levels, it is worth exploring whether it would also be regarded as a predictor to evaluate the economy of ICIs. That would be conducive to filter the appropriate population, thereby avoiding wasting medical resources and enhancing instrumental value. As atezolizumab and pembrolizumab share a similar mechanism, their economic burden and clinical value should be considered by medical decision-makers to facilitate the rational utilization of such expensive schedules.

This study evaluated the cost-effectiveness of atezolizumab and pembrolizumab in comparison with chemotherapy in previously untreated stage IV NSCLC patients, and further sub-analyzed the cost-effectiveness in different PD-L1 expression levels (≥50% and ≥1%). The study was from the perspective of the healthcare system in the US.

## Materials and Methods

### Patients and Interventions

Target population information was derived from two open-label, phase 3, randomized clinical trials (RCTs), IMpower-110 and KEYNOTE-042. Eligible patients were those with histologically or cytologically documented stage IV NSCLC, treatment-naïve, mainly with a known EGFR and ALK translocation wild-type, and had PD-L1 expression at least 1% (4, 5). Due to the lack of head-to-head evidence between two ICIs, an indirect comparison was conducted as the eligible baseline characteristics of these two trials were highly similar. Three first-line treatment regimens were evaluated in this model (1): atezolizumab monotherapy, (2) pembrolizumab monotherapy, and (3) chemotherapy. Patients continued to receive treatment until disease progression or unacceptable toxicity. The drug doses were administered as follows in accordance with trials:

1. Atezolizumab, 1,200 mg intravenously every 3 weeks;
2. Pembrolizumab, 200 mg intravenously every 3 weeks;
3. Platinum-based chemotherapy, every 3 weeks, 4 or 6 cycles, including either cisplatin (75 mg/m^2^) or carboplatin (area under the concentration–time curve [AUC], 6) plus pemetrexed (500 mg/m^2^); cisplatin (75 mg/m^2^) plus gemcitabine (1,250 mg/m^2^) or carboplatin (AUC, 5) plus gemcitabine (1,000 mg/m^2^) intravenously.

After progression, second-line alternative regimens consisted mainly of chemotherapy, targeted therapy, and immunotherapy. Treatment was discontinued after further progression, and from then on, all patients receive the best supportive care (BSC) until death.

### Model Structure

**Figure 1:**
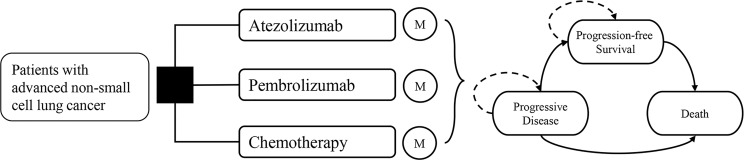
Model structure and transitions.

A Markov model was constructed with a 3-week cycle length and a lifetime horizon. It compares three first-line treatment strategies from the US healthcare system’s perspective. All simulated patients began at progression-free survival (PFS) and then developed into progressive disease (PD) and death, which were all mutually exclusive health states (**Figure 1**).

The primary outcomes of this model were total costs, quality-adjusted life-years (QALYs), and incremental cost-effectiveness ratio (ICER), which was given as the aggregate cost of treatment per QALY gained. A 3% annual discount rate was used, and the willingness-to-pay (WTP) threshold was set as $200,000/QALY in the US setting (6). The model was analyzed using TreeAge Pro 2020 (TreeAge Software, Williamstown, MA).

### Clinical Parameters and Health Utility Inputs

Kaplan–Meier (KM) survival curves used to model PFS and OS were obtained from two original clinical trials (IMpower-110 and KEYNOTE-42) (4, 5). A reliable algorithm derived by Patricia Guyot et al. was applied to obtain reconstructed KM curves and pseudo-individual patient data (IPD) from RCTs reporting time-to-event data (7). Five survival distributions (Weibull, Log-logistic, Log-normal, Gamma, and Exponential) were used to parameterize the curves. The final parametric survival distributions were evaluated based on fit statistics (i.e., Akaike information criterion, AIC) and visual inspection (8). Finally, Weibull distribution was chosen as optimal fitting for two series PFS and OS curves for patients with any PD-L1 expression levels (≥1%) and high PD-L1 expression levels (≥50%) under the trial setting.

QALYs were calculated by combining survival time and health-related quality of life (QOL, a health status value from 0 for death to 1 for perfect health). Health utility values of three strategies were obtained from the relevant pharmacoeconomic studies of NSCLC (9–11).

All the additional information on KM curves and health state utilities are listed in **Table 1**, **Supplementary Figure S1**, and **Supplementary Table S1**.

**Table 1:** Ranges and distributions of parameters used in sensitivity analyses.

Parameters	Base-case values	Min	Max	Distributions	Source
**Drug Costs ($)**					
Atezolizumab (10 mg)	78.28	62.62	93.93	Gamma	CMS
Pembrolizumab (1 mg)	50.84	40.67	61.00	Gamma	CMS
Nivolumab (1 mg)	28.56	22.84	34.27	Gamma	CMS
Carboplatin (50 mg)	2.76	2.21	3.31	Gamma	CMS
Cisplatin (10 mg)	1.71	1.37	2.06	Gamma	CMS
Pemetrexed (10 mg)	71.95	57.56	84.34	Gamma	CMS
Gemcitabine (200 mg)	3.95	3.17	4.75	Gamma	CMS
Paclitaxel (1 mg)	0.15	0.12	0.19	Gamma	CMS
Docetaxel (50 mg)	0.85	0.68	1.02	Gamma	CMS
Bevacizumab (10 mg)	76.36	61.09	91.63	Gamma	CMS
**Treatment costs ($, per cycle)**					
End of life	2,491.08	1,992.97	2,989.30	Gamma	HCUP
BSC	665.33	532.27	798.40	Gamma	(12)
Disease management in PFS	1,482.12	1,185.69	1,778.54	Gamma	(13)
Disease management in PD	1,343.20	1,074.56	1,611.84	Gamma	(13)
Anemia	444.18	429.35	459.01	Gamma	HCUP
Nausea	428.49	402.87	454.11	Gamma	HCUP
Asthenia	665.16	532.13	798.20	Gamma	HCUP
Hyponatremia	330.19	323.51	336.87	Gamma	HCUP
Pneumonia	557.80	446.24	669.36	Gamma	HCUP
Hyperkalemia	324.06	312.85	335.28	Gamma	HCUP
Thrombocytopenia	443.93	408.14	479.72	Gamma	HCUP
Neutropenia	494.25	462.07	526.42	Gamma	HCUP
Febrile neutropenia	596.16	472.12	720.20	Gamma	HCUP
Alanine aminotransferase increased	385.22	365.18	405.26	Gamma	HCUP
**Utility value**					
PFS of Chemotherapy	0.68	0.44	0.92	Beta	(10)
PD of Chemotherapy	0.67	0.47	0.87	Beta	(10)
PFS of Atezolizumab	0.77	0.62	0.92	Beta	(9)
PD of Atezolizumab	0.64	0.51	0.77	Beta	(9)
PFS of Pembrolizumab in any PD-L1 expression	0.69	0.56	0.83	Beta	(11)
PD of Pembrolizumab in any PD-L1 expression	0.47	0.38	0.57	Beta	(11)
PFS of Pembrolizumab in high PD-L1 expression	0.71	0.47	0.95	Beta	(10)
PD of Pembrolizumab in high PD-L1 expression	0.67	0.47	0.87	Beta	(10)
**BSA (m^2^)**	1.82	1.6	2.04	Gamma	(14)
**Body weight (kg)**	70	40	160	Gamma	(15)
**AUC**	6	5	7	Fixed	(3)
**Discount rate (%)**	3	0	8	Fixed	(14)

BSC, best supportive care; PFS, progression-free survival; PD, progressive disease; BSA, body surface area.

### Cost Inputs

The costs were determined from the US healthcare system perspective. Only direct medical costs were considered, including therapeutic drugs, management of treatment-related serious adverse events (SAEs), routine disease administration, BSC, and end-of-life care.

The patients’ body surface area (BSA) and body weight were assumed to be 1.82 m^2^ and 70 kg (14, 15), respectively, to estimate drug dose. The drugs’ prices were derived from the Medicare Part B Drug Spending Dashboard on the Centers for Medicare & Medicaid Services (CMS), which contained the 2020 Drug Average Sales Price in the US (16).

This analysis considered high incidence (≥5%) and grade ≥3 adverse events of the IMpower-110 and KEYNOTE-42 trials. The costs for the management SAEs were collected from the Healthcare Cost and Utilization Project (HCUP) national data based on the 10th Revision of International Classification of Diseases (ICD-10) codes (**Table 1** and **Supplementary Table S2**) (17).

Disease management costs in PFS and PD states referred to hospitalization expenses, laboratory examination fees, and cost of computed tomography and magnetic resonance imaging, which were obtained from previous studies. As the disease progressed further, patients would eventually receive the BSC, and in the final state, patients would accept one-time end-of-life care. The above costs were accessed from previously published research.

All costs were inflated to 2020 value according to the US consumer price index (14, 18).

### Sensitivity Analyses

Univariable deterministic sensitivity analysis (DSA) and probabilistic sensitivity analysis (PSA) were performed to explore the uncertainty in this model. In DSA, all variables varied over a plausible range, set as a variance of 20% parameter value or modified standard deviations to corresponding mean values. Multivariate PSA simultaneously varied model parameters in 1,000 Monte Carlo iterations with a specific pattern of distribution (**Table 1**).

### Budgetary Impact Analysis

A budgetary impact analysis was provided to project the possible additional healthcare expenditure, in which fully implemented ICIs in eligible patients of stage IV NSCLC in the US were assumed. The simulated conditions were matched with those of IMpower-110 and KEYNOTE-42 trials. The total number of eligible patients was estimated based on the Surveillance, Epidemiology, and End Results (SEER) primary database (19). We extracted the annual new cases of patients with NSCLC diagnosed as stage IV, then multiplied by the proportion of PD-L1 expression level based on a published literature (20). As lung cancer incidence is expected to decline in the coming years because of decreasing smoking prevalence in the US, we assumed a 2% decrease in incidence per year (21). The undiscounted costs per year of these two ICIs and chemotherapy strategies were modeled and then the mean difference of annual treatment cost between the more cost-effective ICI and chemotherapy was calculated, multiplied by the number of eligible patients.

## Results

### Base-Case Analysis

The results are shown in **Table 2**. Generally, the ICI monotherapy-gained additional QALYs in the high PD-L1 expression population was evidently longer than that of any PD-L1 expression population, and the costs in the high PD-L1 expression population were correspondingly higher. In any PD-L1 expression population, patients treated with atezolizumab or pembrolizumab provided a gain of 0.47 and 0.23 QALYs over chemotherapy with ICER of $234,990 and $424,797 per QALY, respectively. Atezolizumab was projected to increase 0.24 QALYs and yield an ICER of $56,635/QALY compared with pembrolizumab. In the high PD-L1 expression population, patients treated with atezolizumab or pembrolizumab gained an additional 1.57 and 0.95 QALYs compared with chemotherapy treatment, which led to the ICER of $130,804 and $140,873 per QALY gained, respectively. Besides, compared to pembrolizumab, atezolizumab was also associated with an ICER of $115,511 per QALY.

**Table 2:** Summary of cost and effectiveness results.

Regimen	Chemo	Pembro	Atezo	Incremental		
				Chemo vs. Atezo	Chemo vs. Pembro	Pembro vs. Atezo
**Any PD-L1 expression**						
Total cost ($)	82,258.38	179, 917.3	193,773.7	111,515.32	97,658.91	13,856.37
Total QALYs	0.9499	1.1798	1.4244	0.4746	0.2299	0.2446
ICER per QALY ($)				234,990.23	424,797.1	56,635.92
**High PD-L1 expression**						
Total cost ($)	85,295.8	218,835.8	290,933.7	205,637.9	133,540.00	72,097.9
Total QALYs	0.8948	1.8428	2.4669	1.5721	0.9479	0.6241
ICER per QALY ($)				130,804.59	140,873.27	115,511.82

Chemo, chemotherapy; Pembro, pembrolizumab; Atezo, atezolizumab; QALY, quality-adjusted-life year; ICER, incremental cost-effectiveness ratio.

### Sensitivity Analyses

**Figure 2:**
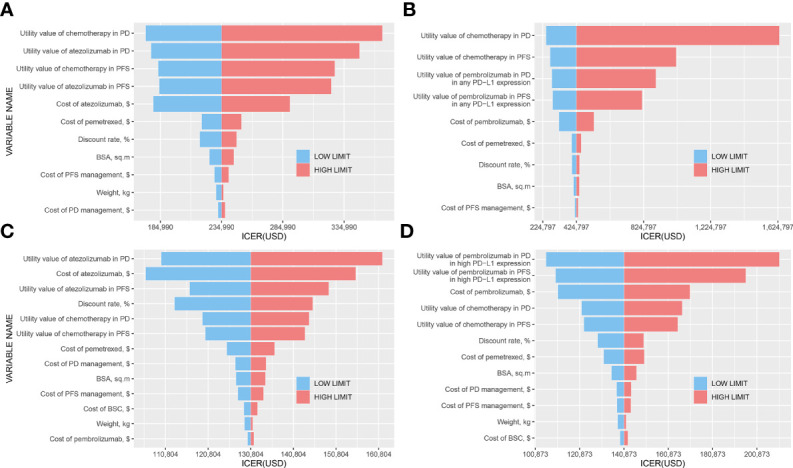
Tornado diagrams. **(A)** Atezolizumab vs. Chemotherapy in any PD-L1 expression population; **(B)** Pembrolizumab vs. Chemotherapy in any PD-L1 expression population; **(C)** Atezolizumab vs. Chemotherapy in high PD-L1 expression population; **(D)** Pembrolizumab vs. Chemotherapy in high PD-L1 expression population.

The tornado diagrams showed some crucial parameters impacting the ICERs remarkably (**Figure 2**). The utility values in PFS and PD states of ICIs and chemotherapy had a significant influence on the ICERs in any PD-L1 expression population, which drove the ICER of atezolizumab vs. chemotherapy to fall below the WTP threshold when a decreased value for each parameter was conducted (**Figures 2A, B**). **Figure 2C** shows that in the high PD-L1 expression level, the utility value of atezolizumab in the PD state and the cost of atezolizumab had the strongest influence on the results. However, the ICER would not exceed $200,000/QALY in upper or lower boundaries of these variants, which indicated the determinacy of results. **Figure 2D** shows that the utility values of pembrolizumab in PFS and PD states were the most influential parameters in high PD-L1 expression level, and thereinto, the ICER might exceed the WTP threshold if we adjusted the utility value in the PD state.

**Figure 3:**
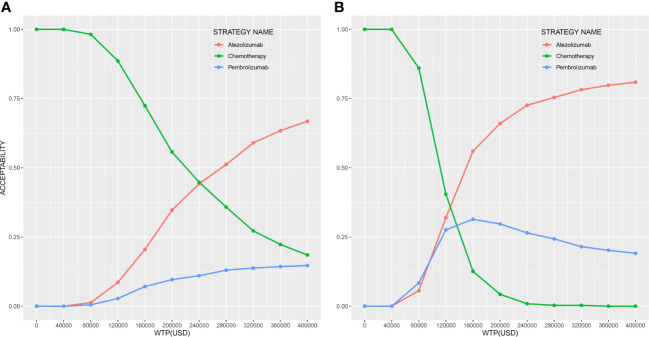
Cost-effectiveness acceptability curves of three strategies **(A)** in any PD-L1 expression population and **(B)** in high PD-L1 expression population.

The results of the PSA are shown in **Figure 3**. Compared to chemotherapy, atezolizumab and pembrolizumab produced probabilities of cost-effectiveness with 34.7% and 9.6% at the WTP threshold in any PD-L1 expression population, and with 66.0% and 29.7% in high PD-L1 expression population, respectively.

### Budgetary Impact Analysis

Owing to the better cost-effective base-case results in atezolizumab when compared with chemotherapy in the high PD-L1 expression population, this further analysis was implemented to estimate the expenditure of using atezolizumab instead of the traditional chemotherapy on patients with high PD-L1 expression patients from 2020 to 2024. The results are presented in **Table 3**. Based on the SEER primary database, we acquired the total number of newly diagnosed stage IV NSCLC, which is 1,312 for 2015, and assumed it as 1,185 for 2020 due to a 2% decrease in lung cancer incidence per year.

**Table 3:** Incremental expenditure ($).

Diagnosed Time	2020	2021	2022	2023	2024
2020	16, 084, 719.60	11, 794, 564.73	8, 127, 814.90	2, 334, 810.82	2, 201, 432.19
2021		15, 763, 025.20	11, 558, 673.44	7, 965, 258.60	3, 121, 787.80
2022			15, 447, 764.70	11, 327, 499.97	7, 805, 953.43
2023				15, 138, 809.41	11, 100, 949.97
2024					14, 836, 033.22
Net budgetary	16, 084, 719.60	27, 557, 589.93	35, 134, 253.03	36, 766, 378.79	39, 066, 156.61

Based on literature data, the proportion of the high PD-L1 expression level was estimated as 22% to determine the number of qualified patients. The net budgetary impact of treating qualified patients with atezolizumab was $32.4 million in the first year and increased rapidly to $60.1 million in the fifth year (**Supplementary Table S3**) (20). By contrast, the net budgetary impact of chemotherapy increased modestly from $16.3 million in the first year to $21.1 million in the fifth year (**Supplementary Table S4**). In the next 5 years, the annual incremental budgetary impact of atezolizumab monotherapy versus chemotherapy would be approximately $16.1 million, $27.6 million, $35.1 million, $36.8 million, and $39.1 million, respectively.

## Discussion

Recently, growing research has been conducted on the cost-effectiveness of ICIs, such as analyses of evaluating the cost-effectiveness of ICIs plus chemotherapy for advanced NSCLC (12, 22, 23). The economic analyses of ICI monotherapy as a first-line treatment regimen for metastatic NSCLC have also been previously evaluated in the US, France, and the UK (10, 24, 25). Currently, a trend of the potential relationship between the PD-L1 expression level and the efficacy of ICIs has been verified by mounting lines of evidence (9, 26, 27). Filtrating the favorable treatment population by using predictive biomarkers would be capable of economizing the gross medical resource, excluding those who are unable to benefit from ICIs. In this way, the detection of PD-L1 expression level could predict clinical benefit, but whether it could be an indicator for cost-effectiveness remains unknown. To our knowledge, this study is the first cost-effectiveness analysis to incorporate the PD-L1 expression levels in comparing two ICIs and chemotherapy in NSCLC.

The base-case results demonstrated that given the WTP threshold of $200,000/QALY in the US healthcare system setting, atezolizumab monotherapy and pembrolizumab monotherapy were more cost-effective treatment strategies compared with chemotherapy in the high PD-L1 expression population. Compared to pembrolizumab, atezolizumab showed notable savings as its ICER ($115,511) was far below the WTP threshold. Meanwhile, the PSA also depicted a higher likelihood that atezolizumab monotherapy would be considered as a cost-effective strategy for high PD-L1 expression level patients at the WTP threshold. Nevertheless, both ICIs were not cost-effective in any PD-L1 expression level populations for stage IV NSCLC. Even so, the ICER of atezolizumab over chemotherapy was much lower than that of pembrolizumab over chemotherapy ($234,990 vs. $424,797), which suggested that the atezolizumab could potentially be more cost-effective. This conclusion was different to a recent cost-effectiveness analysis that combined network-meta analysis conducted in NSCLC patients with PD-L1>50%, which indicated that the first-line cemiplimab monotherapy was a cost-effective option compared with pembrolizumab, while atezolizumab was a minor alternative against cemiplimab or pembrolizumab (28). These two studies were different in the states of model construction, the input parameters (utility values and eligible grade III/IV AEs), as well as the method in calculating the transition probabilities, which might contribute to the discrepancies on the total QALYs and costs and thereby yielded different conclusions.

It is verified that the cost-effectiveness of ICIs compared separately to chemotherapy was identified in the high PD-L1 expression population rather than that in any PD-L1 expression populations owing to the excellent efficacy in the high PD-L1 expression population. The improvement of QALY between atezolizumab and pembrolizumab was more significant in the high PD-L1 expression level population than that in any PD-L1 expression level patients (0.62 QALY vs. 0.24 QALY), so it likewise proved that better effectiveness was provided with atezolizumab in the high PD-L1 expression level. However, the cost-effectiveness result between the two ICIs was not superior in the high PD-L1 expression in comparison with that of any PD-L1 expression population ($115,511 vs. $56,635), probably on account of the substantial expenditure resulted from the longer survival time of atezolizumab. Hence, an applicable predictive biomarker like PD-L1 expression and reliable testing results might be compelling evidence for NSCLC patients when administered atezolizumab or pembrolizumab as the first-line regimen over the long term. Moreover, according to sensitivity results, no matter how one adjusts the cost of these two ICIs in their ranges, the ICERs of the two ICIs compared respectively to chemotherapy in the high PD-L1 expression patients would not exceed the WTP threshold. In that way, if pharmaceutical manufacturers are willing to conduct a medicine price reduction, that could help lighten the financial burden of patients undoubtedly.

As with many other novel cancer medications, ICIs are associated with high expenditure near $10,000 per cycle. Considering the anticipated growth of using ICIs for lung cancer in the coming years, oncologic spending will undoubtedly increase. This incremental budgetary analysis represented a substantial but valuable increase in the overall spending of hypothetical populations with stage IV NSCLC patients receiving atezolizumab monotherapy as a first-line treatment in the US. On the strength of our analysis, the projected incremental budgetary impact was $16.1 million in 2020 and rose to $39.1 million in the coming fifth year. The results indicated that cost savings would not be realized in the next 5 years because of the high number of newly diagnosed patients entering the treated population every year, as well as the exorbitant price of atezolizumab. Cancer treatment has exerted a considerable economic burden on the US healthcare system. According to data from the Agency for Healthcare Research and Quality’s Medical Expenditures Panel Survey for 2016 to 2018, total cancer-related expenditures have increased by a mean of about 6.5% per year (29). At this growth rate, approximately $163 billion will be spent in 2024 on direct medical costs associated with all cancer types. In fact, research has proved that among the 4 most common cancer types (colorectal cancer, lung cancer, breast cancer, and prostate cancer), the highest mean expenditure was in lung cancer patients with $35,141 per patient annually across all age groups (30). Assuming that atezolizumab is used in every qualified advanced lung cancer patient, the total mean treatment expenditures would be roughly estimated as $44.0 million for all new cases for 5 years, which was exceeded by the 5-year atezolizumab treatment expense ($60.1 million) (**Supplementary Table S3**). Such an enormous cost would indeed impose a tremendous burden on the medical financial expenditure of the US.

This study has some strengths, the notable one being the indirect comparison of two ICIs based on the different PD-L1 expression levels. Due to a lack of a head-to-head trial, bare pharmacoeconomic analyses have not been conducted between any ICIs directly yet. Because the eligible baseline characteristics of patients in these two trials (IMpower-110 and KEYNOTE-042) were highly similar, we extracted data from these two trials to compare the cost-effectiveness of two ICIs (atezolizumab and pembrolizumab), which could provide evidence for medical decision-makers when choosing a preferable treatment regime. Besides, the selection of patients with PD-L1 expression ≥ 50% may have a positive impact on the cost-effectiveness of two ICIs, which could facilitate screening the favorable treatment population. In addition, we calculated the budgetary impact analysis of atezolizumab versus chemotherapy in a stage IV NSCLC population based on epidemiological data in the US over the next 5 years. The results can help people who manage health plan budgets to assess the financial impact of adopting new drugs. Similarly, the budgetary impact analysis could be used to enhance the affordability of drugs to patients and the nation as a whole, so as to put forward policy suggestions of price adjustment and offer a proper therapeutic strategy for valid patients. Thus, this measure may boost the cost-effectiveness of drugs like ICIs. Another strength is that the simulated survival data and the pseudo-IPD were generated based on the time to event from KM curves of two RCTs. Compared with other approaches using published KM curves in secondary analysis, the curve reconstruction methods proposed in this model were probably optimal so far, when the least numbers at risk or total events are reported (7). Three curves (original trial survival curves, fitting survival curves, and modeling survival curves) were matched appropriately (**Supplementary Figure S1**), which suggested the excellent accuracy and reliability of our analysis.

The current findings should be considered given the limitations of the analysis. First, because the IMpower110 trial is the only phase III RCT that compared atezolizumab monotherapy with standard chemotherapy in first-line treatment of advanced NSCLC currently (4), in order to provide economic advice on similar types of regimens (atezolizumab and pembrolizumab), we took indirect comparison by integrating two phase III RCTs (4, 5). Both trials were well-designed with feasibly comparable baseline characteristics. Our model was essentially reliant on the validity and generalizability of the trials, but there would be inevitable trivial biases when extrapolating the findings to the real world. Second, owing to the absence of relevant data, the utility values of atezolizumab were obtained from an RCT in which patients received second-line treatment (9, 31). We have assumed a plausible data range on the distribution of the source parameters to ensure the precision and robustness of the model. Third, because the composition ratio of patients with different pathological styles (non-squamous and squamous) had subtle differences in different PD-L1 expression groups (**Supplementary Table S4**), interpreting base-case analysis results between two PD-L1 expression levels should be discreet. Finally, we have generated pseudo-IPD data and used several survival distribution functions to match and extrapolate the curves of trials. However, the long-term benefit of ICIs remains an open question. Further analyses could be conducted based on a large sample size of different PD-L1 expression level subgroups.

## Conclusions

From the US healthcare system perspective, atezolizumab and pembrolizumab are likely to be cost-effective at the WTP threshold compared with standard chemotherapy when used in the first-line setting for stage IV NSCLC patients with high PD-L1 expression. Compared with pembrolizumab, atezolizumab has shown better cost-effectiveness in both populations. The similar efficacy of ICIs across trials suggests that their clinical effectiveness is comparable and dependent on PD-L1 expression levels. Our analysis substantiates that the PD-L1 expression can be considered as a predictor for the cost-effectiveness of ICIs. As ICIs are routinely used gradually, results from this cost-effectiveness analysis will assist future value-based pricing discussions with manufacturers and national subsidy recommendations, which also consider clinical effectiveness, safety, and budget impact.

## Data Availability Statement

The original contributions presented in the study are included in the article/**Supplementary Material**. Further inquiries can be directed to the corresponding authors.

## Author Contributions

XW, XX, and SL contributed to conception and design of the study. DG, PL and LD organized the database. SL and YL performed the statistical analysis. SL and YL wrote the first draft of the manuscript. SHL, TH and PL wrote sections of the manuscript. All authors contributed to manuscript revision, read, and approved the submitted version.

## Funding

This study was supported by the National Natural Science Foundation of China [81973473], the Natural Science Foundation of Fujian Province [2019J01446], and the Startup Fund for Scientific Research, Fujian Medical University [2018QH1091].

## Conflict of Interest

The authors declare that the research was conducted in the absence of any commercial or financial relationships that could be construed as a potential conflict of interest.

## Publisher’s Note

All claims expressed in this article are solely those of the authors and do not necessarily represent those of their affiliated organizations, or those of the publisher, the editors and the reviewers. Any product that may be evaluated in this article, or claim that may be made by its manufacturer, is not guaranteed or endorsed by the publisher.
