# Supplementary material for: The Predictive Value of PD-L1 Expression Level in Evaluating the Cost-Effectiveness of Atezolizumab/Pembrolizumab
Source: Front Oncol. 2022 Apr 22;12:857452. doi: 10.3389/fonc.2022.857452 (PMC9076131; doi:10.3389/fonc.2022.857452)
Supplement: Supplementary file 1 [file Image_1.pdf]

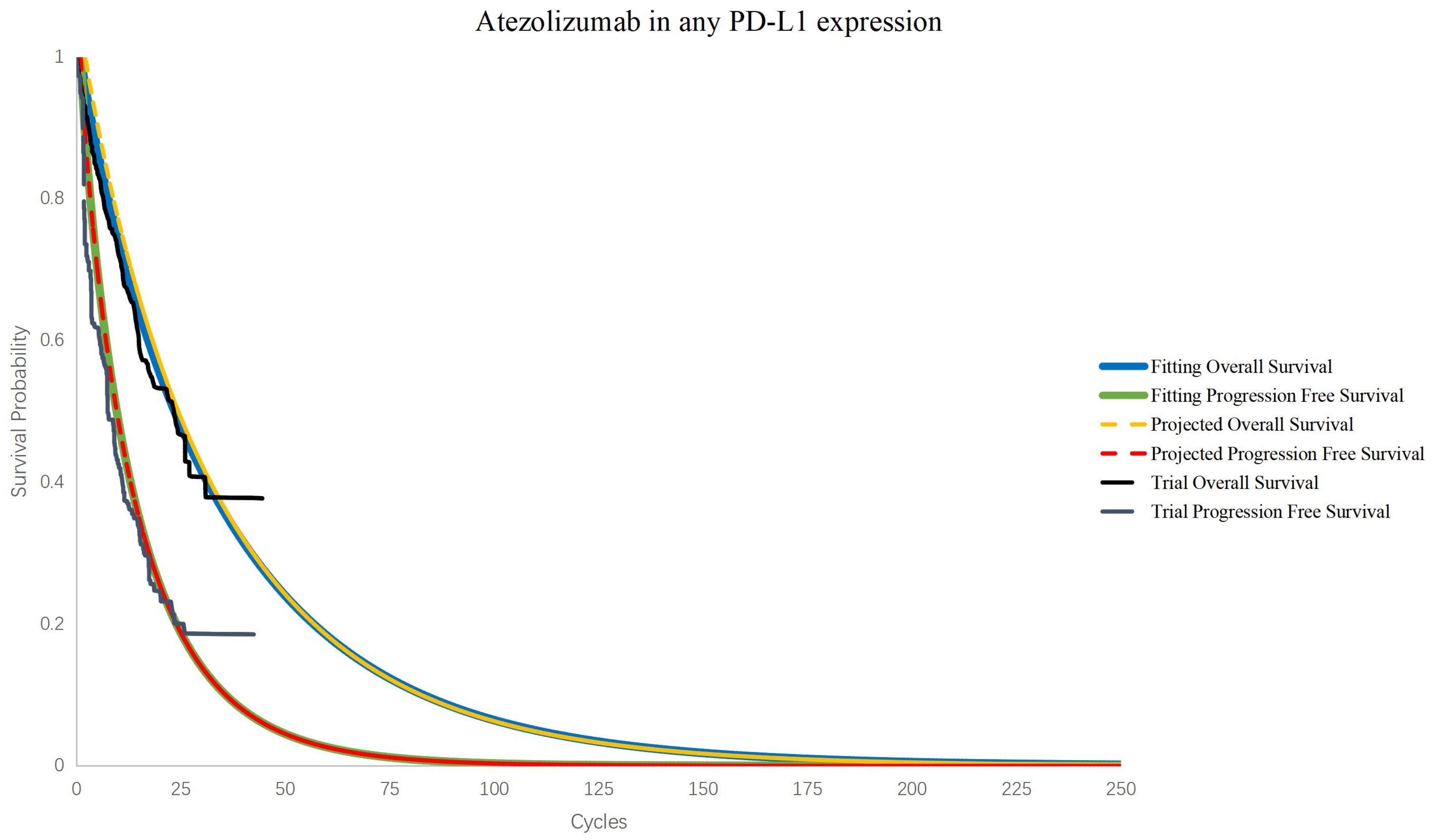
Atezolizumab in any PD-L1 expression
Survival Probability
1
0.8
0.6
0.4
0.2
0
0
25
50
75
100
125
150
175
200
225
250
Cycles
Fitting Overall Survival
Fitting Progression Free Survival
Projected Overall Survival
Projected Progression Free Survival
Trial Overall Survival
Trial Progression Free Survival

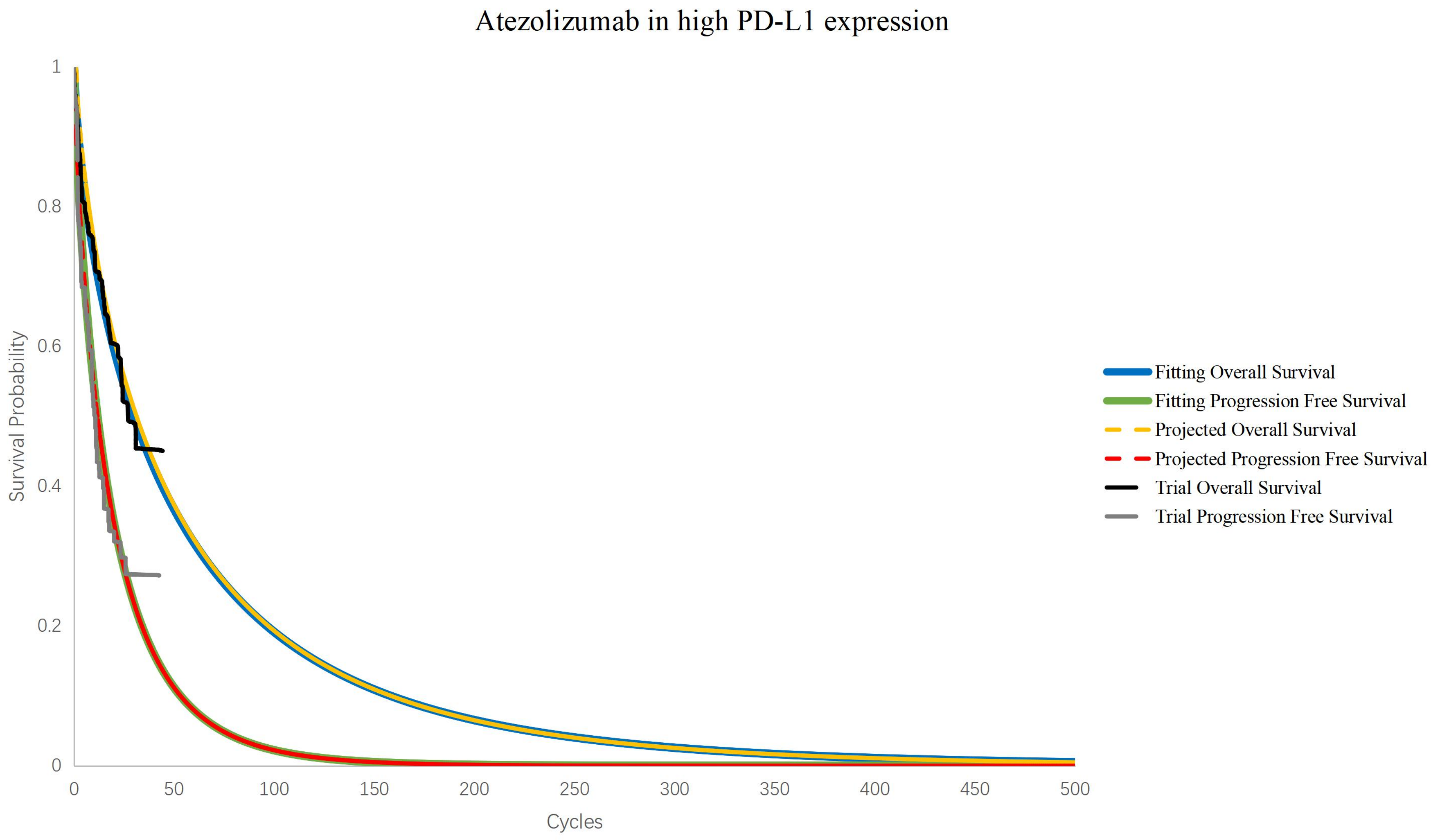

Atezolizumab in high PD-L1 expression
Survival Probability
1
0.8
0.6
0.4
0.2
0
0
50
100
150
200
250
300
350
400
450
500
Cycles
Fitting Overall Survival
Fitting Progression Free Survival
Projected Overall Survival
Projected Progression Free Survival
Trial Overall Survival
Trial Progression Free Survival

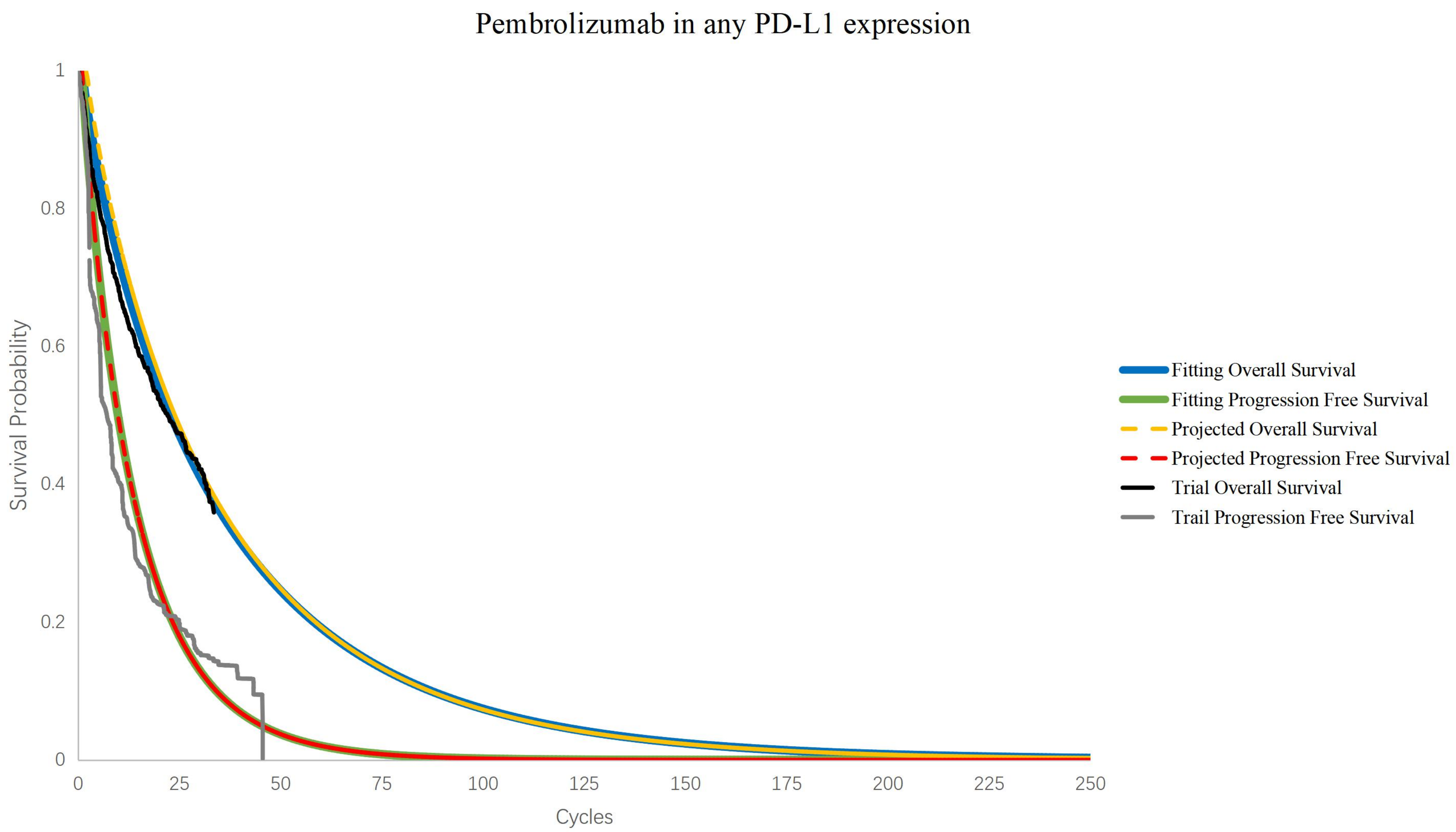
Pembrolizumab in any PD-L1 expression
Survival Probability
1
0.8
0.6
0.4
0.2
0
0
25
50
75
100
125
150
175
200
225
250
Cycles
Fitting Overall Survival
Fitting Progression Free Survival
Projected Overall Survival
Projected Progression Free Survival
Trial Overall Survival
Trail Progression Free Survival

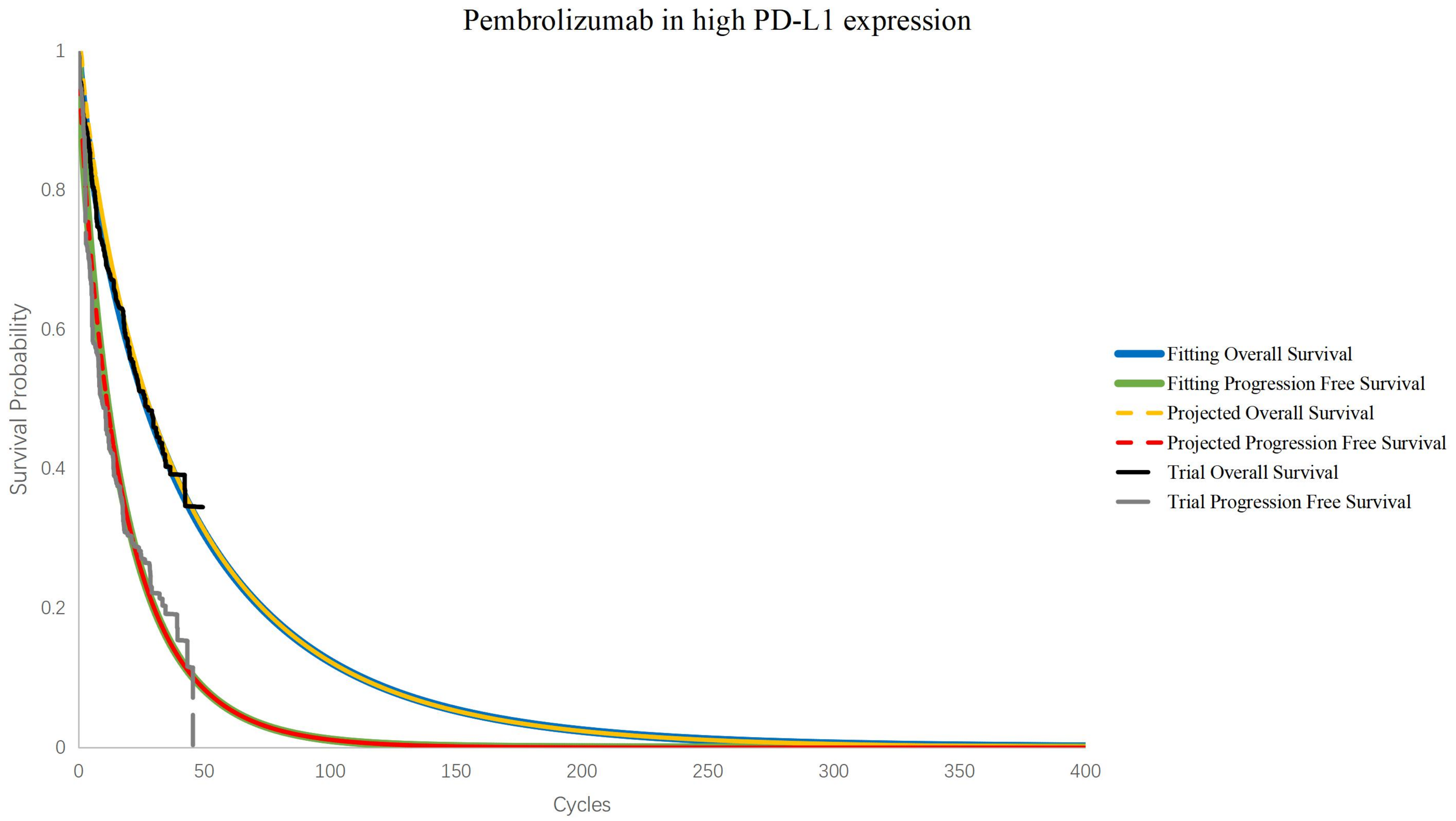
Pembrolizumab in high PD-L1 expression
Survival Probability
1
0.8
0.6
0.4
0.2
0
0
50
100
150
200
250
300
350
400
Cycles
Fitting Overall Survival
Fitting Progression Free Survival
Projected Overall Survival
Projected Progression Free Survival
Trial Overall Survival
Trial Progression Free Survival

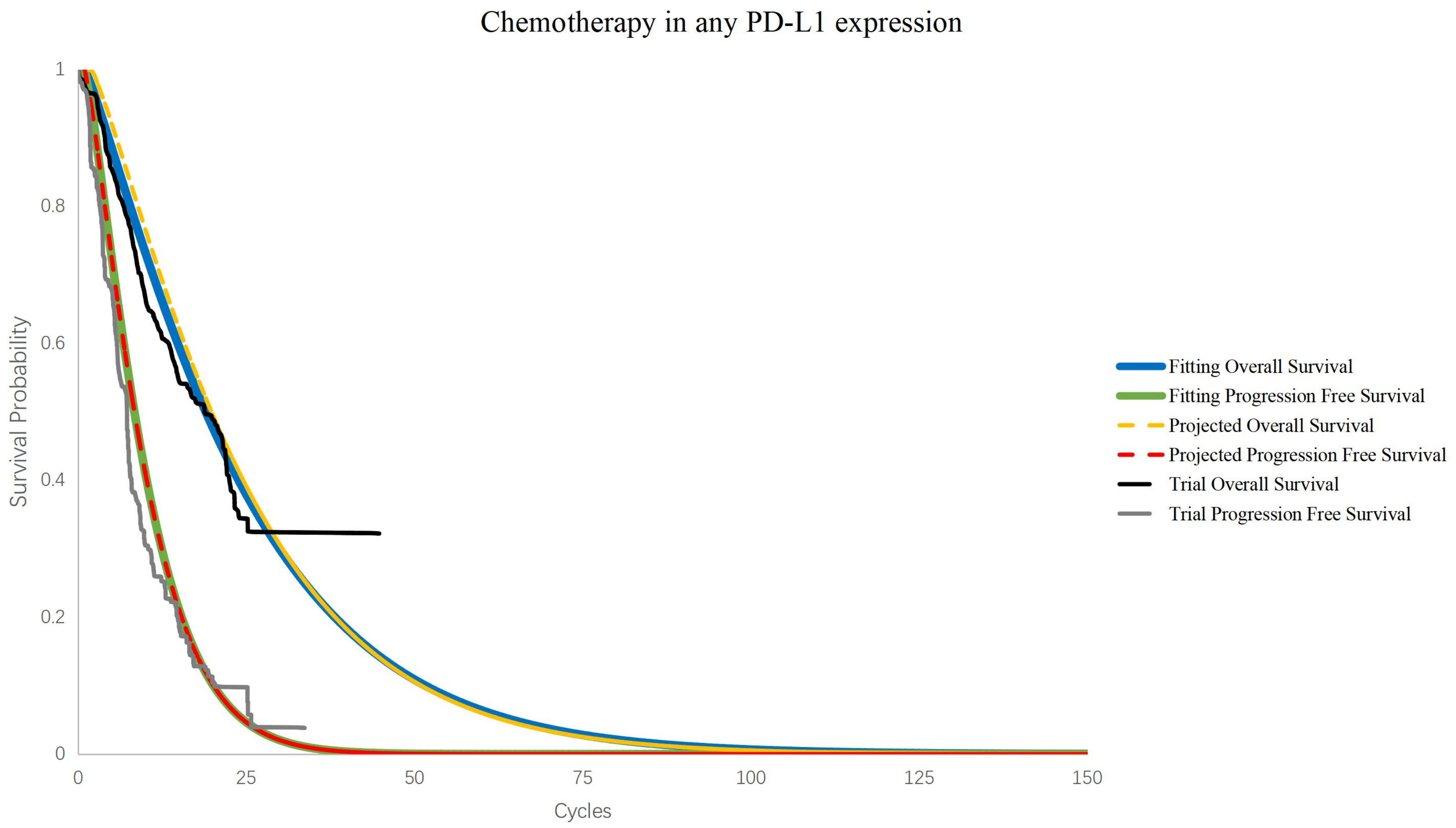
Chemotherapy in any PD-L1 expression
Survival Probability
1
0.8
0.6
0.4
0.2
0
0
25
50
75
100
125
150
Cycles
Fitting Overall Survival
Fitting Progression Free Survival
Projected Overall Survival
Projected Progression Free Survival
Trial Overall Survival
Trial Progression Free Survival

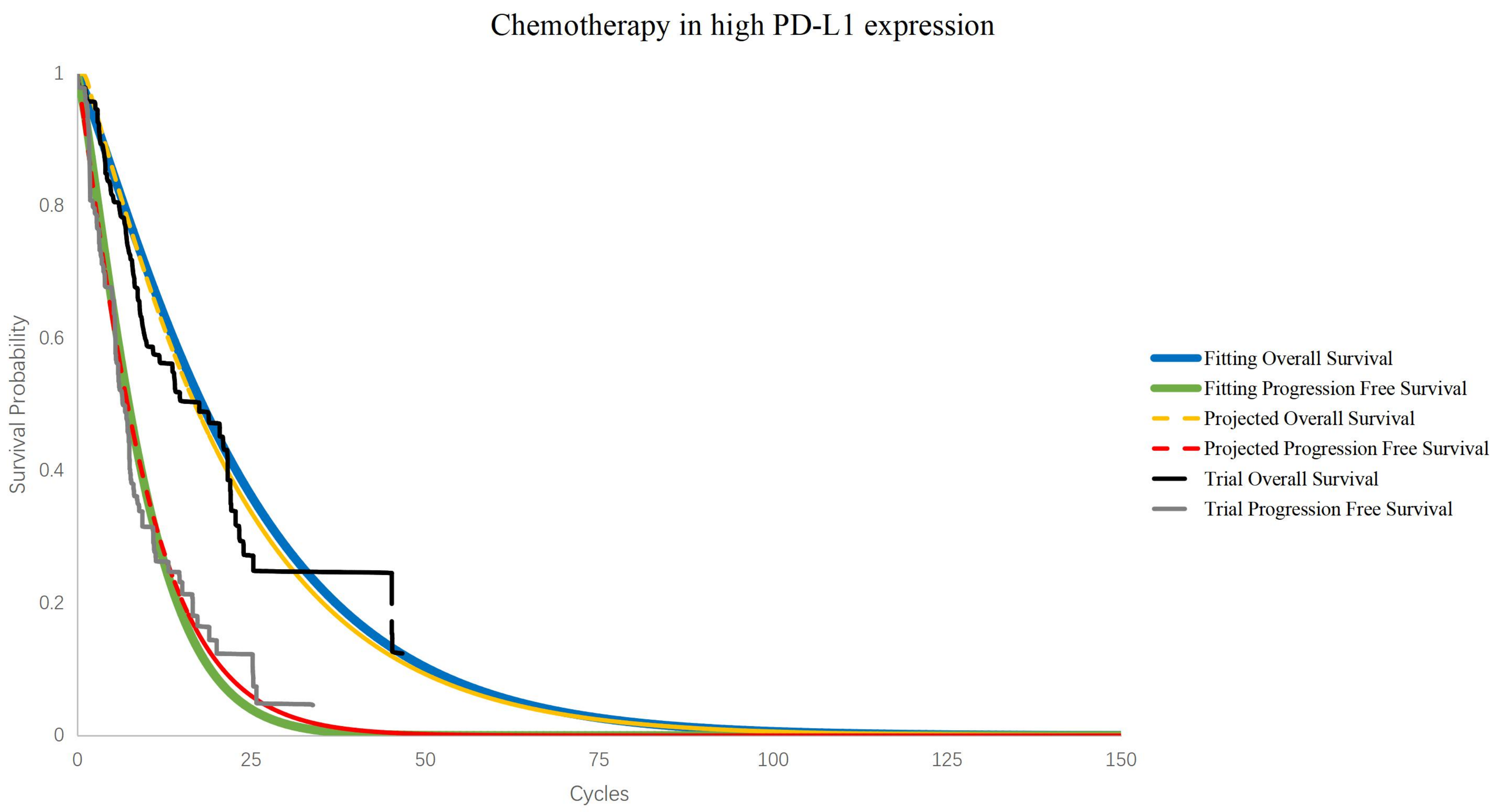

Figure S1 Trial Survival Curves, Fitting Survival Curves and Projected Survival Curves of Three Treatment Strategies
