# Supplementary material for: The Predictive Value of PD-L1 Expression Level in Evaluating the Cost-Effectiveness of Atezolizumab/Pembrolizumab
Source: Front Oncol. 2022 Apr 22;12:857452. doi: 10.3389/fonc.2022.857452 (PMC9076131; doi:10.3389/fonc.2022.857452)
Supplement: Supplementary file 2 [file Table_1.docx]

**Table S1** Distribution Parameters of Survival Curves Fitting

| Strategies | PD-L1 expression level | Distributions | Parameters | PFS | | | OS | | |
| --- | --- | --- | --- | --- | --- | --- | --- | --- | --- |
|  |  |  |  | Est. | AIC | BIC | Est. | AIC | BIC |
| Chemotherapy | high | Loglogistic | shape | 1.617 | 473.8865 | 479.0565 | 1.381 | 442.2185 | 447.3885 |
|  |  |  | scale | 4.887 |  |  | 11.457 |  |  |
|  |  | Weibull | shape | 1.129 | 476.2337 | 481.4036 | 1.079 | 442.9152 | 448.0851 |
|  |  |  | scale | 7.501 |  |  | 17.038 |  |  |
|  |  | Lognormal | meanlog | 1.5625 | 477.9239 | 483.0938 | 2.455 | 448.2195 | 453.3894 |
|  |  |  | sdlog | 1.1162 |  |  | 1.373 |  |  |
|  |  | Gamma | shape | 1.2634 | 475.3437 | 480.5136 | 1.1329 | 442.7519 | 447.9218 |
|  |  |  | rate | 0.1761 |  |  | 0.0681 |  |  |
|  |  | Exponentail | rate | 0.1353 | 475.9875 | 478.5724 | 0.0576 | 441.3755 | 443.9605 |
|  | any | Loglogistic | shape | 1.784 | 1280.290 | 1287.538 | 1.414 | 1058.848 | 1066.096 |
|  |  |  | scale | 5.115 |  |  | 13.202 |  |  |
|  |  | Weibull | shape | 1.2605 | 1284.300 | 1291.548 | 1.1596 | 1060.684 | 1067.932 |
|  |  |  | scale | 7.4090 |  |  | 18.5008 |  |  |
|  |  | Lognormal | meanlog | 1.6021 | 1301.089 | 1308.337 | 2.6275 | 1069.963 | 1077.211 |
|  |  |  | sdlog | 1.0460 |  |  | 1.3439 |  |  |
|  |  | Gamma | shape | 1.4797 | 1281.492 | 1288.740 | 1.2414 | 1060.140 | 1067.388 |
|  |  |  | rate | 0.2133 |  |  | 0.0692 |  |  |
|  |  | Exponentail | rate | 0.1370 | 1298.662 | 1302.286 | 0.05045 | 1062.458 | 1066.082 |
| Pembrolizumab | high | Loglogistic | shape | 1.1755 | 1543.725 | 1551.126 | 0.984 | 1375.939 | 1383.340 |
|  |  |  | scale | 7.2539 |  |  | 19.598 |  |  |
|  |  | Weibull | shape | 0.8518 | 1563.306 | 1570.707 | 0.8277 | 1376.364 | 1383.765 |
|  |  |  | scale | 12.9196 |  |  | 30.7952 |  |  |
|  |  | Lognormal | meanlog | 1.9941 | 1539.588 | 1546.989 | 2.990 | 1377.252 | 1384.653 |
|  |  |  | sdlog | 1.4470 |  |  | 1.821 |  |  |
|  |  | Gamma | shape | 0.84148 | 1567.346 | 1574.747 | 0.79406 | 1377.142 | 1384.543 |
|  |  |  | rate | 0.06212 |  |  | 0.02497 |  |  |
|  |  | Exponentail | rate | 0.07700 | 1570.145 | 1573.845 | 0.03545 | 1381.975 | 1385.675 |
|  | any | Loglogistic | shape | 1.3547 | 3262.002 | 3270.915 | 1.0741 | 3105.278 | 3114.191 |
|  |  |  | scale | 5.6169 |  |  | 15.7155 |  |  |
|  |  | Weibull | shape | 0.9098 | 3335.255 | 3344.168 | 0.8782 | 3111.807 | 3120.721 |
|  |  |  | scale | 9.9536 |  |  | 25.0119 |  |  |
|  |  | Lognormal | meanlog | 1.7553 | 3257.136 | 3266.049 | 2.7526 | 3099.958 | 3108.872 |
|  |  |  | sdlog | 1.2633 |  |  | 1.6177 |  |  |
|  |  | Gamma | shape | 0.94377 | 3341.474 | 3350.387 | 0.86113 | 3114.011 | 3122.925 |
|  |  |  | rate | 0.09257 |  |  | 0.03370 |  |  |
|  |  | Exponentail | rate | 0.09905 | 3340.645 | 3345.102 | 0.04169 | 3118.344 | 3122.801 |
| Atezolizumab | high | Loglogistic | shape | 1.044 | 475.6636 | 481.0093 | 0.831 | 390.4888 | 395.8344 |
|  |  |  | scale | 7.917 |  |  | 23.635 |  |  |
|  |  | Weibull | shape | 0.7956 | 480.6160 | 485.9617 | 0.7229 | 390.6022 | 395.9478 |
|  |  |  | scale | 13.9980 |  |  | 37.4467 |  |  |
|  |  | Lognormal | meanlog | 2.083 | 475.9780 | 481.3236 | 3.269 | 392.3046 | 397.6502 |
|  |  |  | sdlog | 1.678 |  |  | 2.260 |  |  |
|  |  | Gamma | shape | 0.7649 | 482.1845 | 487.5301 | 0.67820 | 390.8816 | 396.2273 |
|  |  |  | rate | 0.0514 |  |  | 0.01737 |  |  |
|  |  | Exponentail | rate | 0.0745 | 484.0516 | 486.7245 | 0.03440 | 395.2798 | 397.9526 |
|  | any | Loglogistic | shape | 1.170 | 1258.184 | 1265.432 | 1.088 | 1020.062 | 1027.310 |
|  |  |  | scale | 5.712 |  |  | 17.085 |  |  |
|  |  | Weibull | shape | 0.8632 | 1273.107 | 1280.355 | 0.9279 | 1021.238 | 1028.486 |
|  |  |  | scale | 9.9093 |  |  | 25.0413 |  |  |
|  |  | Lognormal | meanlog | 1.7551 | 1262.417 | 1269.665 | 2.894 | 1022.796 | 1030.044 |
|  |  |  | sdlog | 1.4975 |  |  | 1.694 |  |  |
|  |  | Gamma | shape | 0.8542 | 1276.067 | 1283.315 | 0.92117 | 1021.482 | 1028.730 |
|  |  |  | rate | 0.0830 |  |  | 0.03669 |  |  |
|  |  | Exponentail | rate | 0.10150 | 1277.609 | 1281.233 | 0.04189 | 1020.144 | 1023.768 |

**Table S2** Cost of Adverse Events According to HCUP

| Adverse Event | Diagnosis/Procedure | ICD-10 Codes | Cost ($, per cycle) |
| --- | --- | --- | --- |
| Anemia | Anemia due to antineoplastic chemotherapy | D64.81 | 444.18 |
| Thrombocytopenia | Other secondary thrombocytopenia | D69.59 | 443.93 |
| Neutropenia | Agranulocytosis secondary to cancer chemotherapy | D70.1 | 494.25 |
| Febrile neutropenia | Fever presenting with conditions classified elsewhere | R50.81 | 596.16 |
| Nausea | Nausea | R11.0 | 428.49 |
| Asthenia | Malaise and fatigue | R53 | 665.16 |
| Hyponatremia | Hypo-osmolality and hyponatremia | E87.1 | 330.19 |
| Pneumonia | Pneumonia, unspecified organism | J18 | 557.80 |
| Hyperkalemia | Hyperkalemia | E87.5 | 324.06 |
| Alanine aminotransferase increased | Nonspecific elevation of levels of transaminase and lactic acid dehydrogenase | R74.0 | 385.22 |

HCUP, Healthcare Cost and Utilization Project, ICD-10, international Classification of diseases-10

All the costs have been adjusted by the consumer price index to the values of 2020.

**Table S3** Budgetary Impact Analysis data ($)

|  | Diagnosed | 2020 | 2021 | 2022 | 2023 | 2024 |
| --- | --- | --- | --- | --- | --- | --- |
| Atezolizumab | 2020 | 32,403,348.16 | 16,005,249.72 | 9,538,151.46 | 3,716,940.65 | 2,407,984.99 |
|  | 2021 |  | 31,755,281.20 | 15,685,144.72 | 9,347,388.43 | 3,642,601.84 |
|  | 2022 |  |  | 31,120,175.57 | 15,371,441.83 | 9,160,440.66 |
|  | 2023 |  |  |  | 30,497,772.06 | 15,064,012.99 |
|  | 2024 |  |  |  |  | 29,887,816.62 |
|  | Net budgetary | 32,403,348.16 | 47,760,530.92 | 56,343,471.76 | 58,933,542.97 | 60,162,857.1 |
| Chemotherapy | 2020 | 16,318,628.57 | 4,210,684.90 | 1,410,336.57 | 531,442.90 | 206,552.80 |
|  | 2021 |  | 15,992,255.99 | 4,126,471.29 | 1,382,129.83 | 520,814.03 |
|  | 2022 |  |  | 15,672,410.87 | 4,043,941.86 | 1,354,487.24 |
|  | 2023 |  |  |  | 15,358,962.66 | 3,963,063.03 |
|  | 2024 |  |  |  |  | 15,051,783.4 |
|  | Net budgetary | 16,318,628.57 | 20,202,940.98 | 21,209,218.73 | 21,316,477.24 | 21,096,700.5 |
| Incremental Cost | 2020 | 16,084,719.60 | 11,794,564.73 | 8,127,814.90 | 2,334,810.82 | 2,201,432.19 |
|  | 2021 |  | 15,763,025.2 | 11,558,673.44 | 7,965,258.60 | 3,121,787.80 |
|  | 2022 |  |  | 15,447,764.70 | 11,327,499.97 | 7,805,953.43 |
|  | 2023 |  |  |  | 15,138,809.41 | 11,100,949.97 |
|  | 2024 |  |  |  |  | 14,836,033.22 |
|  | Net budgetary | 16,084,719.60 | 27,557,589.93 | 35,134,253.03 | 36,766,378.79 | 39,066,156.61 |

**Table S4** Main population characteristic of two clinical trials

|  | Atezolizumab | | Pembrolizumab | | Chemotherapy | |
| --- | --- | --- | --- | --- | --- | --- |
|  | PD-L1 ≥1% | PD-L1 ≥50% | PD-L1 ≥1% | PD-L1 ≥50% | PD-L1 ≥1% | PD-L1 ≥50% |
| Age | 64 (30–81) | 63 (33–79) | 63.0 (57.0-69.0) | 63.0 (56.0–68.0) | 65 (30–87) | 66 (33–87) |
| Men | 70.8% | 73.8 | 71% | 69% | 69.7% | 65.3% |
| ECOG performance status score | | | | | | |
| 0 | 35.0% | 32.7% | 31.0% | 32.0% | 36.8% | 38.8% |
| 1 | 65.0% | 67.3% | 69.0% | 68.0% | 63.2% | 61.2% |
| Tumor histological features | | | | | | |
| Squamous | 30.7% | 25.2% | 38.0% | 36.0% | 30.3% | 23.5% |
| Non-squamous | 69.3% | 74.8% | 62.0% | 64.0% | 69.7% | 76.5 |
